# Retrieval practice is costly and is beneficial only when working memory capacity is abundant

**DOI:** 10.1038/s41539-023-00159-w

**Published:** 2023-03-31

**Authors:** Yicong Zheng, Pengyuan Sun, Xiaonan L. Liu

**Affiliations:** 1 Department of Psychology, University of California, Davis, CA USA; 2 Center for Neuroscience, University of California, Davis, CA USA; 3 Department of Psychology, National University of Singapore, Singapore, Singapore; 4 Department of Psychology, The Chinese University of Hong Kong, Hong Kong, Hong Kong

**Keywords:** Human behaviour, Education

## Abstract

Numerous studies have shown that learned information practiced by testing is better retained than that practiced by restudying (the testing effect). However, results are inconsistent regarding the effect of working memory (WM) capacity on the testing effect. Here, we hypothesize that the effect of WM only emerges when task demands challenge WM capacity. We manipulated WM demands by pretraining 30 undergraduate participants in a multi-session visual search task before an associative learning task involving a test/restudy manipulation. The results revealed that, while participants with higher WM capacity showed a consistent testing effect, the benefit of testing only emerged in participants with lower WM capacity when learning familiar stimuli (low WM demands). We simulated the results using a modified source of activation confusion (SAC) model, which implemented a dual-process account of the testing effect. The results suggested that the testing effect only emerges when WM capacity is adequate for both processes.

## Introduction

Testing can not only be used to assess studying but also improve learning and enhance later retention, a phenomenon widely known as the *testing effect* (refs. ^1–4^, see^5^ for a meta-analytic review). The prototypical paradigm of the testing effect^1^ involves an initial learning phase, a practice phase, in which half of the learned items are studied again (i.e., restudy) and the other half tested (i.e., retrieval practice), and a final test phase that evaluates memory retention for all items. The typical finding is that in the final test, items practiced in the test condition are recalled better than those in the restudy condition.

While the testing effect has been shown to be a robust learning technique in education^6^, it is less clear whether testing should be uniformly applied in the classroom and whether testing helps certain subpopulations more than others. Several studies have suggested that the magnitude of the testing effect might be affected by individual differences, such as working memory (WM) capacity. For example, Tse & Pu^7^ found an interaction between WM capacity and test anxiety, such that test anxiety only reduced the testing effect in participants with lower WM capacity. In other words, people with a lower WM capacity may benefit less from the testing effect in certain scenarios. However, this effect was not replicated by a study using a similar paradigm but different materials that are in participants’ native language^8^. Indeed, there is no consensus on how WM capacity affects the testing effect. Several studies have found that WM capacity has no significant relationship to the testing effect and the forward testing effect, which refers to the effect that testing old information can improve the learning of new information^9–13^.

One factor that might moderate the relationship between WM capacity and the testing effect is whether feedback (i.e., correct answer) is given after retrieving. For example, one study found that participants with lower WM capacity benefits more from retrieval practice than those with higher WM capacity, but this effect only emerged when feedback was given^14^. Another study found that older adults (usually with lower WM capacity) learned better through restudying than through retrieval practice when no feedback was provided; however, the testing effect emerged when feedback was provided^15^. These studies suggest that WM capacity only moderates the testing effect under more difficult conditions (e.g., no feedback). The reason for the abovementioned inconsistent results might be that some experiments did not challenge individuals’ WM limits, especially for those with higher WM capacity.

Based on these results, we conjecture that the benefit of testing is moderated by both available WM resources and WM demands during retrieval practice. Specifically, prior research suggests that the testing effect involves separate contributions from a retrieval attempt process and a post-retrieval re-encoding process^16–18^. Both processes consume WM resources (e.g., maintenance of retrieval cues, active search for targets, and maintenance of retrieved information;^19,20^). When WM demands of the task are high, participants with lower WM capacity may have used all available WM resources after the retrieval attempt process and thus benefit little from the re-encoding process. In contrast, when WM demands are low, even participants with lower WM capacity may benefit from both processes.

**Fig. 1:**
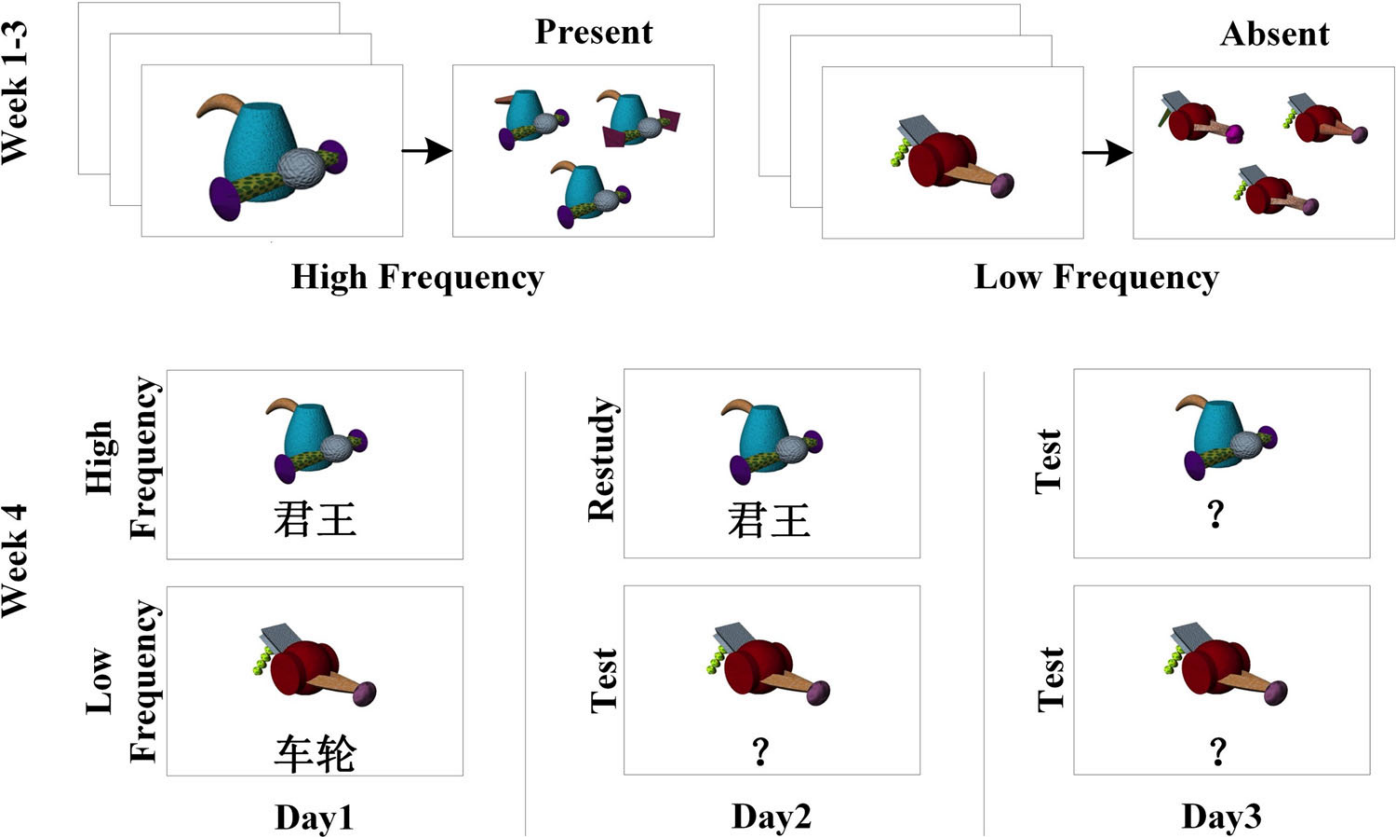
Experiment procedure. *Top panel*: Example trials of the visual search task during the first three weeks. The presentation ratio of High Frequency: Low Frequency was 15:1. *Bottom panel*: Procedure of the associative learning task, including the initial learning (left panel), the retrieval/restudy practice (middle panel), and the final test (right panel).

In this study, in addition to measuring individual WM capacity, we manipulated WM demands by varying the frequency of novel stimuli through three weeks of pretraining in an adaptation of the training paradigm from Reder et al.^21^. Reder et al.^21^ showed that in an associative learning task, high-frequency (HF) stimuli required fewer WM, and low-frequency (LF) stimuli required more WM resources to form new associations. In the fourth week, participants learned randomly formed associations of HF or LF stimuli and practiced through retrieval practice or restudy (Fig. 1). We predict that if WM resources are abundant, the testing effect will emerge regardless of the WM demands of the stimuli. In contrast, for participants with limited WM capacity, the testing effect will only emerge when WM demands are low. Beyond the experiment, we simulated the two processes involved in retrieval practice^16–18^ by adapting the source of activation confusion (SAC) model^22^, which assumes that learning and retrieving new information in long-term memory consumes WM resources from a limited pool.

## Results

### The testing effect is modulated by the interaction between frequency and WM capacity

We examined the testing effect as a function of individual WM capacity and stimulus frequency. Individual WM capacity scores were calculated using the d’ values (${{{{\rm{d}}}}}^{{\prime} }={{{{\rm{Z}}}}}_{Hit}-{{{{\rm{Z}}}}}_{FalseAlarm}$) of the 2-back and 3-back tasks. The 1-back blocks were not included because participants only needed to compare the current stimulus with the immediately preceding one, and the accuracy was close to 100%. We fitted three mixed-effects logistic regression models (Table 1): a main-effect model, a model with two-way interactions, and a model with a three-way interaction. In our models, the fixed effects were Frequency (HF vs. LF), Condition (test vs. restudy), WM capacity, and interactions. The random effect was the participant-specific intercept. The main-effect model revealed a significant testing effect (*b* = 0.23, *z* = 2.29, *p* = 0.022) and a significant main effect of WM capacity (*b* = 0.51, *z* = 2.32, *p* = 0.020). Accuracy was higher for participants with higher WM capacity. Importantly, the results revealed a three-way interaction between Condition, Frequency, and WM capacity (*b* = −0.83, *z* = −2.44, *p* = 0.015).

**Table 1:** Regression coefficients of the main effect model (Model 1), two-way interaction model (Model 2), and three-way interaction model (Model 3).

Model	Parameter	*b*	*β*	*z*	*p*	95% CI (*β*)
1	(Intercept)	−0.77	0.39	−1.47	0.141	[0.11, 0.68]
	Condition	0.23*	0.23	2.29	0.022	[0.03, 0.42]
	Frequency	0.15	0.15	1.50	0.134	[−0.05, 0.34]
	WM	0.51*	0.30	2.32	0.020	[0.05, 0.55]
2	(Intercept)	−0.56	0.45	−0.96	0.336	[0.15, 0.75]
	Condition	−0.34	0.12	−0.83	0.405	[−0.15, 0.40]
	Frequency	0.18	0.04	0.45	0.654	[−0.23, 0.31]
	WM	0.44	0.26	1.78	0.074	[−0.03, 0.55]
	Condition * Frequency	0.23	0.23	1.14	0.255	[−0.16, 0.62]
	Condition * WM	0.20	0.12	1.19	0.233	[−0.08, 0.32]
	Frequency * WM	−0.06	−0.04	−0.38	0.707	[−0.23, 0.16]
3	(Intercept)	−0.12	0.45	−0.20	0.843	[0.14, 0.75]
	Condition	−1.27*	0.13	−2.27	0.023	[−0.14, 0.41]
	Frequency	−0.71	0.04	−1.30	0.194	[−0.23, 0.32]
	WM	0.25	0.15	0.96	0.338	[−0.15, 0.45]
	Condition * Frequency	2.10**	0.20	2.65	0.008	[−0.19, 0.60]
	Condition * WM	0.62*	0.36	2.56	0.011	[0.08, 0.64]
	Frequency * WM	0.33	0.20	1.41	0.157	[−0.08, 0.47]
	Condition * Frequency * WM	−0.83*	−0.49	−2.44	0.015	[−0.88, −0.10]

*p < 0.05; **p < 0.01.

**Fig. 2:**
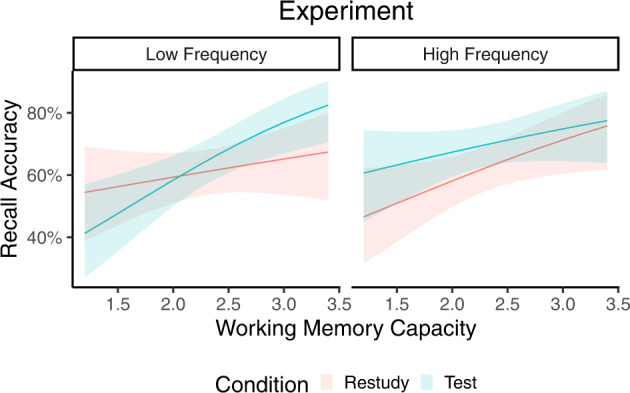
Predictions of the final test accuracy based on the mixed-effects logistic regression models. While all participants showed the testing effect in HF associations, only participants with higher WM capacity showed the testing effect in LF associations. The shaded areas represent the 95% confidence intervals for the fitted regression curves.

Follow-up analyses revealed a significant testing effect in the main-effect model (*b* = 0.48, *z* = 2.43, *p* = 0.015), but no significant two-way interaction effect in HF associations (Table 2). In contrast, there was a significant Condition by WM interaction in LF associations (*b* = 0.61, *z* = 2.55, *p* = 0.011, Table 3). As shown in Fig. 2, participants with different WM capacities exhibited different magnitudes of testing effects in LF associations. This pattern was confirmed by a simple slopes analysis, which showed that participants with higher WM capacity (+ 1 SD) exhibited a significant testing effect (*b* = 0.50, *z* = 2.43, *p* = 0.015), whereas participants with lower WM capacity (−1 SD) exhibited a trend towards a negative testing effect (*b* = −0.22, *z* = −1.15, *p* = 0.249, Fig. 2).

**Table 2:** Regression coefficients of the main effect model (Model 1) and two-way interaction model (Model 2) for HF associations.

Model	Parameter	*b*	*β*	*z*	*p*	95% CI (*β*)
1	(Intercept)	−0.60	0.49	−1.01	0.310	[0.17, 0.81]
	Condition	0.35*	0.35	2.43	0.015	[0.07, 0.63]
	WM	0.48	0.28	1.92	0.055	[−0.01, 0.57]
2	(Intercept)	−0.85	0.49	−1.31	0.191	[0.17, 0.81]
	Condition	0.87	0.34	1.52	0.129	[0.06, 0.62]
	WM	0.59*	0.35	2.13	0.034	[0.03, 0.67]
	Condition * WM	−0.23	−0.14	−0.94	0.347	[−0.42, 0.15]

*p < 0.05.

**Table 3:** Regression coefficients of the main effect model (Model 1) and two-way interaction model (Model 2) for LF associations.

Model	Parameter	*b*	*β*	*z*	*p*	95% CI (*β*)
1	(Intercept)	−0.79	0.52	−1.41	0.159	[0.24, 0.79]
	Condition	0.12	0.06	0.84	0.400	[−0.08, 0.20]
	WM	0.55*	0.32	2.30	0.021	[0.05, 0.60]
2	(Intercept)	−0.13	0.52	−0.21	0.832	[0.24, 0.80]
	Condition	−1.26*	0.07	−2.26	0.024	[−0.07, 0.21]
	WM	0.26	0.33	0.97	0.332	[0.05, 0.61]
	Condition * WM	0.61*	0.18	2.55	0.011	[0.04, 0.32]

*p < 0.05.

Because there was no feedback in the test condition on Day 2, one might wonder whether the effect of WM capacity was driven by the possibility that participants with lower WM capacity might have recalled fewer LF associations than HF associations. In comparison, participants with higher WM capacity might have recalled a similar amount of HF and LF associations. While we had controlled this potential confound by applying the same learning criteria for LF and HF associations on Day 1, to further exclude this possibility, we performed the same regression while controlling for the accuracy ratio between HF and LF associations during retrieval practice (see Supplementary Notes for details). The results exhibited the same pattern.

### Modified SAC explained the relationship between WM and the testing effect

**Fig. 3:**
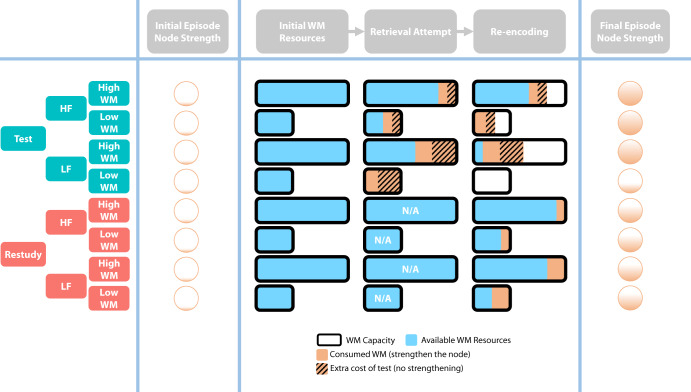
Schematic diagram illustrating how the modified SAC model predicts the outcome of each condition in the current experiment. We assume that both the retrieval attempt and post-retrieval re-encoding processes consume WM resources (brown and brown with diagonal lines) from a limited WM pool (i.e., WM capacity (black frame)), resulting in left WM resources (blue) for future use. The post-retrieval re-encoding process only strengthens the nodes when retrieval attempts are successful because no feedback is provided. The consumption of WM negatively correlates with the Fribble frequency (HF for high frequency, LF for low frequency) and is used to strengthen the episode node, as depicted in the final column. When the available WM resources (blue) are not sufficient for both processes (i.e., participants with lower WM capacity learning LF associations), the associations cannot benefit from the post-retrieval re-encoding process, thus limiting the overall testing effect.

To account for the effects of WM on the processes underlying the testing effect, the SAC model^22^ was adapted, incorporating the dual-process (retrieval attempt and post-retrieval re-encoding) hypothesis of the testing effect^16–18^. A diagram of the modified SAC model is shown in Fig. 3 to demonstrate how the modified SAC predicts the final episodic memory strength given: 1) condition (test/restudy), 2) frequency (HF/LF), and 3) WM capacity (high/low).

**Fig. 4:**
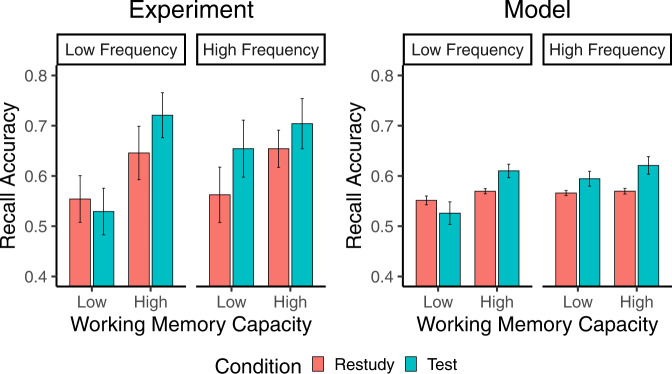
The simulation results captured the critical three-way interaction in the experiment. While all participants benefited from testing in HF associations, only participants with higher WM capacity showed the testing effect in LF associations. Error bars represent standard errors of the mean.

In Fig. 4, both experimental and simulation results were broken down into separate bars by a median split of WM capacity to better illustrate the interaction between WM capacity, stimulus frequency, and the testing effect. The simulation results captured the critical patterns of the experimental results. Specifically, the key finding of a WM capacity by condition interaction in LF associations was replicated. Participants with lower WM capacity showed no significant testing effect, but a trend toward a negative testing effect in LF associations. In comparison, participants with higher WM capacity consistently exhibited the testing effect.

## Discussion

The current study showed that the testing effect was moderated by both individual WM capacity and WM demands, as determined by the nature of the stimuli. Specifically, people with abundant WM resources benefited from retrieval practice regardless of the stimulus frequency. In contrast, people with lower WM resources only showed the testing effect in HF associations and a trend toward a negative testing effect in LF associations. We adapted the SAC model^22^ based on the dual-process hypothesis of the testing effect^16–18^ to account for our findings. The simulation results exhibited the same pattern as the experimental results.

An important contribution of the current study is reconciling the conflicting results regarding the effects of WM on the testing effect^7,9–13^. The results demonstrated that retrieval practice is a costly learning technique, but the bottleneck of WM only emerges when WM demands challenge WM capacity. Specifically, our results suggest that one possible explanation for prior studies that did not find an effect of WM capacity on the testing effect is the lack of WM demands in the study design. For example, the inconsistent findings of the anxiety by WM capacity interaction between Tse & Pu^7^ and Tse et al.^8^ might be reconciled by the current model. Specifically, if word pairs in a foreign language^7^ required more WM resources than knowledge facts in the native language^8^, individuals with lower WM capacity would be expected to show a tendency of decreased testing effect when learning word pairs in a foreign language and the WM capacity effect may be signified by test anxiety. Moreover, the current model can explain the results that older participants only benefit from retrieval practice when feedback was provided but learned better through restudying when no feedback was provided^15^. One possibility is that feedback serves as a WM aid that reduces WM demands, thus increasing the testing effect for those with lower WM capacity. Future studies may be conducted to examine the role of feedback in participants with different WM capacities.

The study also shed light on the underlying mechanisms of the testing effect. The results support the dual-process hypothesis of the testing effect^16–18^. Specifically, if the testing effect involves only the benefits of the retrieval attempt per se, memories should be fully strengthened as long as the correct targets are retrieved. Thus, the magnitude of the testing effect should be a function of retrieval practice accuracy. Because both HF and LF associations were learned to the same criteria, a single process account would predict comparable magnitudes of the testing effect in HF and LF associations. Moreover, because we controlled for individual differences in the accuracy ratio between HF and LF associations in retrieval practice, a single process account would predict no moderating effect of individual differences. In other words, a single-process account would not predict the three-way interaction among WM capacity, frequency, and condition.

In contrast, the two-process hypothesis argues that, in addition to the retrieval attempt process, participants also benefit from re-encoding correctly retrieved information (Fig. 3). The current results, including the three-way interaction, were fully replicated by our simulation (Fig. 4), which implemented the dual-process hypothesis in the well-established SAC model^22^. Simply put, the critical assumption of the model is “trading” WM resources for memory strengthening, such that people have their WM resource pools of different sizes and improve memorization by spending these resources. Because the re-encoding process occurs after the correct answer is obtained, successfully recalled associations may not be fully strengthened if WM resources are depleted during the retrieval attempt. Thus, LF associations are less likely to benefit from retrieval practice when challenging individuals’ WM capacity.

It is noteworthy that the power of this study is relatively small due to the difficulty of recruiting participants for the month-long experiment. We did not have an estimation of the effect size for the key interaction between WM capacity, Frequency, and Condition. Therefore, the a priori power analysis was conducted to ensure the sample size was adequate to detect the testing effect. We conducted a sensitivity power analysis with the current sample size using the summary-statistics-based power analysis^23^. The results showed that for the critical three-way interaction, the current sample size was adequate to detect a medium to large effect size (*r* > 0.485) according to Cohen (1992). Future studies with a larger sample size are needed to detect smaller effects.

In summary, the current study showed that the effect of WM on the testing effect only emerges when the task challenges WM capacity. The experiment and our simulation suggest that successfully retrieving the item does not guarantee that the memory is more effectively strengthened than restudying the item. The testing effect involves a post-retrieval process that further strengthens memory. While this study supports the hypothesis that two temporally distinct processes are involved in the testing effect, the cognitive mechanisms underlying these processes remain unclear. One possibility is that the two processes reflect learning in the hippocampus and neocortex, as described in a recent biologically realistic neural network model^24^. Future work may examine how the two temporally distinct processes map onto learning mechanisms in different neural networks.

## Methods

### Participants

Thirty-five undergraduate students from Xiamen University participated in the experiment. The sample size was determined by an a priori power analysis using the summary-statistics-based power analysis for mixed-effects modeling of nested data^23^. Based on the t-value for the testing effect in our prior study, using the same materials and a similar procedure^25^, 33 participants were required to achieve 80% power. All participants had normal or corrected-to-normal vision and spoke Chinese as their first language. Two participants were excluded because of low accuracy in the final test (below three standard deviations from the mean). Two participants were excluded because of low performance (d’ less than 1) on the 1-back task. One participant was excluded due to data loss. All participant exclusions were performed before running the data analyses and modeling. Thirty participants were included in the final analyses and computational modeling of the experiment. The final sample size achieved 77% power. The study was approved by the Ethics Committee of Xiamen University. All participants gave written informed consent in accordance with the Declaration of Helsinki.

### Materials

We used novel animal-like objects, named “Fribbles”^26^, as cues to form associations. Each Fribble consists of a body and four appendages, providing varying degrees of visual similarity across objects. The use of Fribbles controlled for potential prior experiences with the stimuli. Sixty-four Chinese nouns were selected as targets from the Chinese Corpus database (http://corpus.zhonghuayuwen.org/) and randomly paired with Fribbles to form cue-target associations.

### Procedure

The design was adapted from Reder et al.^21^ and lasted for 4 weeks for each participant (Fig. 1). During the first three weeks, participants were pretrained to familiarize themselves with the Fribbles in a visual search task, with half of the Fribbles presented more frequently than the other half. After the pretraining, participants performed an associative learning task over three consecutive days in the final week. After the initial learning on Day 1, half of the associations were studied again, while the other half were tested without feedback on Day 2. The final test of all associations was conducted on Day 3. The procedure for each task is described in detail below:

#### Visual search task

The participants completed a visual search task to familiarize themselves with 64 Fribbles over nine sessions over three weeks. Among all the Fribbles, half (32) were randomly selected to be presented at high frequency (HF), while the other half (32) were presented at low frequency (LF). Each trial showed a target Fribble, followed by three similar Fribbles that shared the same main body. Participants were asked to indicate whether the target Fribble was present (50% of the trials) or absent (50% of the trials) from the three alternative Fribbles. The ratio between HF and LF was 15:1 (i.e., 32 HF Fribbles were presented 15 times as frequently as the 32 LF Fribbles). The interval between the two sessions in the same week was 24–48 h.

#### N-back task

After the third training session each week, the participants performed an N-back task in which stimuli were drawn from Fribbles from the HF and LF conditions. In addition to measuring WM capacity, this task allowed us to test our hypothesis that more familiar stimuli consume fewer WM resources. Each Fribble was shown for 2000 ms on the screen, and participants were required to decide whether the current stimulus matched the stimulus presented N pictures before. In this task, N varied from 1 to 3 in different blocks. Half of the blocks used Fribbles from the HF condition, and the other half used Fribbles from the LF condition. There were four blocks for each of the six conditions (2 frequency conditions × 3 N-back levels), and each block contained 18 trials. The order of the blocks was randomized for each participant.

#### Day 1 initial learning

On Day 1 of the final week, participants performed an associative learning task in which 64 Fribbles (32 HF + 32 LF) pretrained during the first three weeks were randomly associated with 64 new Chinese two-character words. During each trial, after the presentation of a fixation cross for 1000 ms, a Fribble and a word were simultaneously displayed on the top and bottom of the screen, respectively, for 8000 ms. After learning all of the associations, the participants were tested on the associations for multiple rounds. During the test trial, participants were cued with a Fribble and asked to type the two-letter initials of the associated words. Feedback was provided after the participants entered their answers. An association was dropped from subsequent testing rounds if it was correctly recalled in two consecutive rounds. The testing rounds ended after all the associations were dropped. This procedure ensured that all associations were learned to the same degree after Day 1.

#### Day 2 restudy/retrieval practice and Day 3 final test

On Day 2 (24 h after Day 1), participants first studied all of the associations again, in the same format as the Day 1 learning, to refresh their memory. After the refresh phase, half of the associations (16 HF + 16 LF) were assigned to the test condition, while the other half (16 HF + 16 LF) were assigned to the restudy condition. Participants were tested on or restudied assigned associations in randomized and intermixed order. In a test trial, after a 1000 ms fixation cross, a Fribble was first shown in the center of the screen for 4000 ms. The participants were then encouraged to recall the associated word. On the next screen, the participants were presented with the Fribble on the left and a question mark on the right. Participants were asked to type the initial letters of the associated word within 10 s. Feedback was not provided after each trial. In a restudy trial, a Fribble and the target word were presented simultaneously for 4000 ms. Participants were asked to enter the initial letters of the target word within the following 10 s. On Day 3 (24 h after Day 2), all 64 Fribble-word associations were tested once in the same format as the test trials on Day 2.

### Statistics

We used mixed-effects logistic regression^27,28^ to examine how WM capacity and stimulus frequency influence the testing effect. The mixed-effects logistic regression models were fitted using the “lme4” package^29^ in the R Statistical Environment^30^. Simple slopes analyses were performed following significant interactions using the “interactions” package^31^.

### Model simulation

#### Model description

The main idea underlying the SAC model^22^ is that the components of episodic memory can be represented as nodes in a localist network, and activation of one node will spread to connected nodes. These nodes can be broadly categorized as “concept” or “episode,” such that multiple concept nodes (e.g., contexts and items) can connect to a common episode note. The strength of a memory trace is described as the base-level strength and activation level, indicating the quality of the memory stored in the brain and the level of current activation during retrieval, respectively. Nodes activated by either proactive retrieval or the spread of activation from connected nodes will have a high temporary activation level, increasing the base-level strength. Note that these two indices of memory strength decay over time.

In addition, the SAC model accounts for the interplay between long-term memory and WM. Specifically, it hypothesizes that individuals have limited WM resource pools, such that people with higher WM will have more WM resources to use during various tasks and vice versa. Furthermore, node operations (e.g., encoding and retrieval) deplete WM resources, which are gradually replenished as a function of time. Because the default recovery rate allows the WM capacity pool to replenish fully in a few seconds, WM capacity in the SAC model only affects memory formation at the trial level. This assumption is consistent with the study showing that retrieval practice does not compete for WM resources with subsequent arithmetic tasks after a longer delay^32^. The familiarity of nodes influences the usage of WM resources during operations. For instance, if an item has never been seen before, encoding and retrieving it will consume more WM resources than items that are highly familiar to participants. Note that the core principles of SAC directly link item familiarity to node strength (i.e., repeatedly experiencing a node enhances familiarity and node strength).

Besides SAC principles, we incorporated three new assumptions. Specifically, 1) we assumed that retrieval practice involves two sequential processes: retrieval attempt and post-retrieval re-encoding (of retrieved information). This assumption is supported by our previous fMRI and EEG studies which have identified two temporally distinct processes that contribute to the testing effect^16–18^. 2) We used the default learning rate in the SAC model for the two processes. According to the SAC model, in the critical condition that WM resources are not adequate for both processes, memory strengthening is determined by available WM resources but not the learning rate. Therefore we used the same learning rate for simplicity and consistency with the SAC model. 3) We assumed that retrieval practice consumes extra WM resources that do not directly strengthen memory, such as memory search that does not lead to the correct target, as well as maintaining the retrieved information. This assumption is proposed to account for the trend in the current experiment that individuals with lower WM capacity performed better in the restudy condition compared to the test condition. The extra WM resource consumption was assumed to be inversely proportional to the cue and target nodes’ base-level strengths, following Eq. (1):

1 $${{{{\rm{WM}}}}}_{extra}={{{{\rm{w}}}}}_{e}(1/{{{{\rm{B}}}}}_{cue}+1/{{{{\rm{B}}}}}_{target}),$$

Where WM_*extra*_ is the requested extra WM resources, w_*e*_ is the weighting parameter to be fitted, and B_*cue*_ and B_*target*_ are the base-level strengths of the paired cue fribble and the target word, respectively.

#### Simulation procedure

All Fribbles and words in the experiment were defined as concept nodes. Because we did not manipulate the context of the experiment, we added a quasi-context node for all associations for simplicity. The initial base-level strengths of the 64 fribble nodes (32 HF + 32 LF) were calculated by applying the simulated familiarization procedure during the first three weeks to the memory strength delay formula in SAC^22^. Specifically, we discretized the 135 repetitions of HF stimulus presentations and 9 repetitions of LF stimuli over nine days across three weeks, maintaining the HF: LF ratio at 15:1. After decaying, the initial base-level strengths of the HF and the LF nodes were 0.91 and 0.46, respectively.

We simplified the simulation to include only Days 2 and 3 of the final week because the same criteria of associative learning were achieved on Day 1. Therefore, we assumed that the simulation started by refreshing all of the associations once and then going through randomly intermixed restudy/test trials as in the experiment. Because there was no feedback provided for test trials, the post-retrieval re-encoding process strengthened the episode node only if the retrieval attempt was successful. After 24 h of decay, the model was tested by performing a cued recall task.

The predicted accuracy for each trial was continuously transformed from the activation level of the episode node using the cumulative distribution function (CDF) of the normal distribution specified by the mean *θ* and standard deviation *σ*. Both *θ* and *σ* were estimated at the individual level. In addition, we fitted the w_*e*_ value at the group level, which determined the extra WM cost during retrieval, as specified in Eq. (1). The d’ values of the N-back task were used as individual WM capacities in the model. First, we looped through each possible w_*e*_ with a precision of 0.01. We used this value and each participant’s WM capacity to estimate individual *θ* and *σ* by minimizing the root-mean-square error between the predicted accuracy and the observed accuracy across the four conditions (2 frequencies × 2 conditions). Next, we averaged the *θ* and *σ* values across participants to calculate the group-level root-mean-square error between the predicted and observed accuracy in these four conditions. The best w_*e*_ value was determined by minimizing the group-level error. The averaged *θ* and *σ* corresponding to the w_*e*_ were used to define the CDF. Except for the three estimated parameters, all other parameters were inherited from the SAC model^22^, as shown in Supplementary Table 1.

### Reporting summary

Further information on research design is available in the Nature Research Reporting Summary linked to this article.

### Supplementary information

Supplementary Material

Reporting Summary

## Data Availability

Data generated during the study and simulations used in the current study are publicly available at https://osf.io/thu36/?view_only=0defb68380ef469cb144a77aedeb5d46.

## Code availability

Scripts of the computational model are publicly available at https://osf.io/thu36/?view_only=0defb68380ef469cb144a77aedeb5d46.
