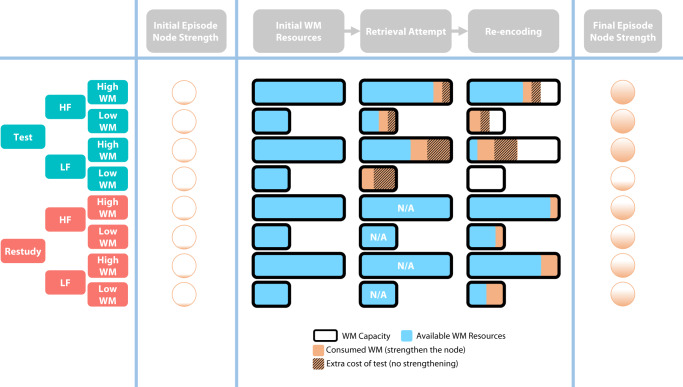# Publisher Correction: Retrieval practice is costly and is beneficial only when working memory capacity is abundant

**DOI:** 10.1038/s41539-023-00185-8

**Published:** 2023-09-07

**Authors:** Yicong Zheng, Pengyuan Sun, Xiaonan L. Liu

**Affiliations:** 1 Department of Psychology, University of California, Davis, CA USA; 2 Center for Neuroscience, University of California, Davis, CA USA; 3 Department of Psychology, National University of Singapore, Singapore, Singapore; 4 Department of Psychology, The Chinese University of Hong Kong, Hong Kong, Hong Kong

**Keywords:** Human behaviour, Education

Correction to: *npj Science of Learning* 10.1038/s41539-023-00159-w, published online 31 March 2023

In the PDF of this article the wrong figure appeared as Fig. 3; the figure should have appeared as shown below. The PDF has been corrected.