# Supplementary material for: Retrieval practice is costly and is beneficial only when working memory capacity is abundant
Source: NPJ Sci Learn. 2023 Mar 31;8:8. doi: 10.1038/s41539-023-00159-w (PMC10066312; doi:10.1038/s41539-023-00159-w)
Supplement: Supplementary file 1 — Supplementary Material [file 41539_2023_159_MOESM1_ESM.pdf]

Supplementary Information

Supplementary Table

Supplementary table 1: Model parameter descriptions

Model Parameter	Description	Value
d_n	Power decay rate for node base-level strength	-0.18
d_l	Power decay rate for link strength	-0.12
y	Exponential decay rate for current activation	0.20
δ	Learning rate for base-level strength	0.80
w_r	WM recovery rate	0.97
W	Total WM resource capacity	Individual N-back d-prime
θ^*	Retrieval threshold	0.40
σ^*	Standard deviation of the noise added to activation	0.50
w_e^*	Weight for extra WM demands during retrieval practice	0.17

All parameters were inherited from the original SAC model except W, which took individual N-back d-prime values from the experiment. Note: Asterisks indicate parameters that were fitted to these data. The others were imported from the original SAC model.

Supplementary Notes

To rule out the possibility that the effect of WM capacities was confounded by individual differences in retrieval practice accuracy ratio between HF and LF associations, we ran the same regression while controlling for the accuracy ratio between HF and LF associations during retrieval practice (formula: ACC $\sim$ HLRatio + Condition + Frequency + WM + HLRatio * Condition + HLRatio * Frequency + Condition * Frequency + Condition * WM + Frequency * WM + HLRatio * Fre-

quency * Condition + Condition * Frequency * WM). The model also included participants as the random effect (formula: ~ 1 | participant).

Results showed the same pattern as the main text results. There was a significant three-way interaction (b = -0.93, z = -2.47, p = 0.014). No significant two-way interaction was found in HF associations. However, there was a significant WM by Condition interaction in LF associations. Simple slopes analysis suggested that participants with higher WM (+1 SD) showed a significant testing effect (b = 0.52, z = 2.43, p = 0.015), while participants with lower WM (-1 SD) showed no significant testing effect (b = -0.25, z = -1.23, p = 0.221).
