# Supplementary material for: Retrieval practice is costly and is beneficial only when working memory capacity is abundant
Source: NPJ Sci Learn. 2023 Mar 31;8:8. doi: 10.1038/s41539-023-00159-w (PMC10066312; doi:10.1038/s41539-023-00159-w)
Supplement: Supplementary file 2 — Reporting Summary [file 41539_2023_159_MOESM2_ESM.pdf]

nature portfolio

Corresponding author(s): Xiaonan L. Liu

Last updated by author(s): Mar 8, 2023

Reporting Summary

Nature Portfolio wishes to improve the reproducibility of the work that we publish. This form provides structure for consistency and transparency in reporting. For further information on Nature Portfolio policies, see our Editorial Policies and the Editorial Policy Checklist.

Statistics

For all statistical analyses, confirm that the following items are present in the figure legend, table legend, main text, or Methods section.

n/a	Confirmed	
☐	☒	The exact sample size (*n*) for each experimental group/condition, given as a discrete number and unit of measurement
☐	☒	A statement on whether measurements were taken from distinct samples or whether the same sample was measured repeatedly
☐	☒	The statistical test(s) used AND whether they are one- or two-sided<br>*Only common tests should be described solely by name; describe more complex techniques in the Methods section.*
☐	☒	A description of all covariates tested
☐	☒	A description of any assumptions or corrections, such as tests of normality and adjustment for multiple comparisons
☐	☒	A full description of the statistical parameters including central tendency (e.g. means) or other basic estimates (e.g. regression coefficient) AND variation (e.g. standard deviation) or associated estimates of uncertainty (e.g. confidence intervals)
☐	☒	For null hypothesis testing, the test statistic (e.g. *F*, *t*, *r*) with confidence intervals, effect sizes, degrees of freedom and *P* value noted<br>*Give P values as exact values whenever suitable.*
☒	☐	For Bayesian analysis, information on the choice of priors and Markov chain Monte Carlo settings
☒	☐	For hierarchical and complex designs, identification of the appropriate level for tests and full reporting of outcomes
☐	☒	Estimates of effect sizes (e.g. Cohen's *d*, Pearson's *r*), indicating how they were calculated

Our web collection on statistics for biologists contains articles on many of the points above.

Software and code

Policy information about availability of computer code

Data collection	Eprime 3.0
Data analysis	R Studio (2022.02.3)

For manuscripts utilizing custom algorithms or software that are central to the research but not yet described in published literature, software must be made available to editors and reviewers. We strongly encourage code deposition in a community repository (e.g. GitHub). See the Nature Portfolio guidelines for submitting code & software for further information.

Data

Policy information about availability of data

All manuscripts must include a data availability statement. This statement should provide the following information, where applicable:

- Accession codes, unique identifiers, or web links for publicly available datasets
- A description of any restrictions on data availability
- For clinical datasets or third party data, please ensure that the statement adheres to our policy

Data generated during the study and simulations used in the current study are publicly available at https://osf.io/thu36/?view_only=0defb68380ef469cb144a77aedeb5d46

Human research participants

Policy information about studies involving human research participants and Sex and Gender in Research.

Reporting on sex and gender	Sex and gender are not considered in the study design.
Population characteristics	See below
Recruitment	Participants were recruited from undergraduate students in Xiamen University
Ethics oversight	The study was approved by the Ethical Committee of Xiamen University

Note that full information on the approval of the study protocol must also be provided in the manuscript.

Field-specific reporting

Please select the one below that is the best fit for your research. If you are not sure, read the appropriate sections before making your selection.

☐ Life sciences ☒ Behavioural & social sciences ☐ Ecological, evolutionary & environmental sciences

For a reference copy of the document with all sections, see nature.com/documents/nr-reporting-summary-flat.pdf

Behavioural & social sciences study design

All studies must disclose on these points even when the disclosure is negative.

Study description	This is an experimental study in psychology, we investigated long term memory and its interaction with working memory. All collected data are quantitative.
Research sample	After exclusion of data, we had a sample of 30 undergraduate students (18-22 years old) from Xiamen University.
Sampling strategy	Sampling was random. Based on the t-value for the testing effect in our prior study, using the same materials and a similar procedure, 33 participants were required to achieve 80% power.
Data collection	All data were recorded and collected via computer. The researchers who conducted this study were not blind to the experimental condition and to the study aims.
Timing	10/11/2017-12/27/2018
Data exclusions	Two participants were excluded because of low accuracy in the final test (below three standard deviations from the mean). Two participants were excluded because of low performance (d' less than 1) on the 1-back task. One participant was excluded due to data loss. All participant exclusions were performed before running the data analyses and modeling.
Non-participation	No participants dropped out or declined participation.
Randomization	Participants were not allocated into experimental groups.

Reporting for specific materials, systems and methods

We require information from authors about some types of materials, experimental systems and methods used in many studies. Here, indicate whether each material, system or method listed is relevant to your study. If you are not sure if a list item applies to your research, read the appropriate section before selecting a response.

Materials & experimental systems

n/a	Involved in the study
☒	☐ Antibodies
☒	☐ Eukaryotic cell lines
☒	☐ Palaeontology and archaeology
☒	☐ Animals and other organisms
☒	☐ Clinical data
☒	☐ Dual use research of concern

Methods

n/a	Involved in the study
☒	☐ ChIP-seq
☒	☐ Flow cytometry
☒	☐ MRI-based neuroimaging
